# High-Frequency Variability of Bacterioplankton in Response to Environmental Drivers in Red Sea Coastal Waters

**DOI:** 10.3389/fmicb.2022.780530

**Published:** 2022-03-31

**Authors:** Mohd Ikram Ansari, Maria LI. Calleja, Luis Silva, Miguel Viegas, David Kamanda Ngugi, Tamara Megan Huete-Stauffer, Xosé Anxelu G. Morán

**Affiliations:** ^1^Division of Biological and Environmental Sciences and Engineering (BESE), Red Sea Research Center (RSRC), King Abdullah University of Science and Technology (KAUST), Thuwal, Saudi Arabia; ^2^Department of Biosciences, Integral University, Lucknow, India; ^3^Department of Climate Geochemistry, Max Planck Institute for Chemistry (MPIC), Mainz, Germany; ^4^Department of Microorganisms, Leibniz Institute DSMZ - German Collection of Microorganisms and Cell Cultures, Braunschweig, Germany; ^5^Centro Oceanográfico de Gijón/Xixón (IEO, CSIC), Gijón/Xixón, Spain

**Keywords:** bacterioplankton, seasonality, flow cytometry, 16S rDNA, next-generation sequencing, single-cell analysis, DOC, Red Sea

## Abstract

Autotrophic and heterotrophic bacterioplankton are essential to the biogeochemistry of tropical ecosystems. However, the processes that govern their dynamics are not well known. We provide here a high-frequency assessment of bacterial community dynamics and concurrent environmental factors in Red Sea coastal waters. Weekly sampling of surface samples during a full annual cycle at an enclosed station revealed high variability in ecological conditions, which reflected in changes of major bacterioplankton communities. Temperature varied between 23 and 34°C during the sampling period. Autotrophic (*Synechococcus*, 1.7–16.2 × 10^4^ cells mL^−1^) and heterotrophic bacteria (1.6–4.3 × 10^5^ cells mL^−1^) showed two maxima in abundance in spring and summer, while minima were found in winter and autumn. Heterotrophic cells with high nucleic acid content (HNA) peaked in July, but their contribution to the total cell counts (35–60%) did not show a clear seasonal pattern. Actively respiring cells (CTC+) contributed between 4 and 51% of the total number of heterotrophic bacteria, while live cells (with intact membrane) consistently accounted for over 90%. Sequenced 16S rRNA amplicons revealed a predominance of Proteobacteria in summer and autumn (>40%) and a smaller contribution in winter (21–24%), with members of the Alphaproteobacteria class dominating throughout the year. The contribution of the Flavobacteriaceae family was highest in winter (21%), while the Rhodobacteraceae contribution was lowest (6%). Temperature, chlorophyll-*a*, and dissolved organic carbon concentration were the environmental variables with the greatest effects on bacterial abundance and diversity patterns.

## Introduction

Prokaryotes are the dominant life forms in the oceans and primary components of aquatic food webs. They play fundamental roles in ecosystem functioning and biogeochemical processes at local and global scales (Azam et al., 1983; Falkowski et al., 2008; DeLong, 2009; Sarmento et al., 2010; Bunse and Pinhassi, 2017). Both autotrophic (i.e., cyanobacteria of the genera *Synechococcus*, *Prochlorococcus*, *Trichodesmium*, and other diazotrophs) and heterotrophic bacteria and archaea are also affected by anthropogenic changes (Allison and Martiny, 2008; Buttigieg et al., 2018). Cyanobacteria are key contributors to primary production and nitrogen fixation, especially in oligotrophic regions (Björkman et al., 2015; Rahav et al., 2016; Liang et al., 2017), while heterotrophic bacterioplankton are ubiquitous and assimilate a large fraction of labile dissolved organic carbon (DOC) and convert it back to CO_2_ (Del Giorgio et al., 1997; James et al., 2017). Flow cytometric studies allow differentiation and enumeration of heterotrophic bacterioplankton cells into different groups, such as low nucleic acid (LNA) and high nucleic acid (HNA) cells, (living cells with intact membranes; Bouvier et al., 2007), or actively respiring cells (CTC+; Gasol et al., 1999; Bouvier et al., 2007). Previous studies have shown that HNA and CTC+ bacterial cells are metabolically more active (Baltar et al., 2015), and that respiration, especially of these groups, is closely related to bulk bacterial production (del Giorgio et al., 1997; Longnecker et al., 2005).

Seasonal succession of microbial taxa have been demonstrated in time-series studies (Giovannoni and Vergin, 2012; Szabo et al., 2012; Teeling et al., 2016), including the gross flow cytometric groups of LNA and HNA cells (Morán et al., 2015), although some authors have not found temporal patterns across bacterioplankton communities due to lack of sharp transitions (Gilbert et al., 2012; Hatosy et al., 2013; Chafee et al., 2017). However, time-series with frequent sampling revealed more pronounced changes in the relative abundance of microbial taxa (Lindh et al., 2015; Needham and Fuhrman, 2016; Martin-Platero et al., 2018), and variability in cell-specific characteristics can be compared to variation in the entire bacterioplankton assemblage due to environmental factors (del Giorgio and Gasol, 2008; Sjöstedt et al., 2018). The effects of environmental factors such as temperature, inorganic and organic nutrient concentrations, or dissolved oxygen levels on bacterial community composition and metabolic response may allow for short- and long-term ecological variation. How the effects of these changes on marine microbes impact ecosystem function is a challenging research topic (Pinhassi and Hagström, 2000; Fuhrman et al., 2006; Alonso-Saez et al., 2008; Bunse and Pinhassi, 2017).

Temporal changes can be very rapid, as shown in mesocosm experiments with high-frequency sampling that show variations in bacterial community abundance and composition on hourly, daily, and weekly time scales (van Hannen et al., 1999; Schäfer et al., 2001; Schauer et al., 2003; Chu et al., 2018). In coastal waters, external forcing such as freshwater runoff, rainfall, dust deposition, or anthropogenic pressure (Khatri and Tyagi, 2015; Bojarczuk et al., 2018) can cause rapid changes in the biogeochemical properties of the system. These factors can trigger a sudden succession in the bacterioplankton community until it recovers and returns to its previous composition (Yeo et al., 2013). Seasonal variability of bacterioplankton in tropical environments has been studied in only a few locations. However, seasonality in different oceanic environments has been well studied with inter-annual time-series (Giovannoni and Vergin, 2012; Cram et al., 2015). For example, the coastal waters of Qinhuangdao in northern China show distinct seasonal patterns in bacterioplankton abundance in cold and warm seasons (He et al., 2017).

The Red Sea, an enclosed, relatively undisturbed, primarily tropical, narrow, and deep marine basin, is one of the hottest marine regions on Earth. It is an oligotrophic sea with limited inorganic nitrogen and phosphorus concentrations because of almost no riverine inputs and very little precipitation. In parallel to strong latitudinal gradients in salinity and surface temperature (increasing and decreasing, respectively, northwards), inorganic nutrients show also a marked north–south gradient (Ngugi et al., 2012; Berumen et al., 2019). Replenishment mostly comes from the Indian Ocean, making the north and central parts very oligotrophic in comparison (Sofianos et al., 2002).

Consequently, the surface waters of the Red Sea are dominated by the picoplankton size-class, with *Prochlorococcus* and SAR 11 being the most abundant autotrophic and heterotrophic groups, respectively (Ngugi et al., 2012). Evidence based on metagenomics shows that both functional and microbial diversity covary with physicochemical parameters, with temperature explaining about half of the variation (Thompson et al., 2017). Other studies show that environmental conditions (e.g., temperature, chlorophyll *a*, oxygen, and particulate organic matter concentrations) shape prokaryotic community composition in surface waters of the northeastern half of the Red Sea (Ngugi et al., 2012). However, these results are based on one-time samplings conducted mainly in open waters. Thus, knowledge about temporal changes in bacterioplankton abundance and community composition in Red Sea coastal waters is still unresolved. Recently, Silva et al. (2019) described the monthly variability in specific growth rates and maximum attainable abundances of heterotrophic bacteria in the harbor area of King Abdullah University of Science and Technology (KAUST). Sabbagh et al. (2020) reported for the same place the weekly changes in autotrophic and heterotrophic bacteria abundance as well as their top-down controls, viruses and heterotrophic flagellates. These studies concluded that the low total abundances of heterotrophic bacterioplankton did not preclude them for showing high growth rates and were rather caused by strong mortality year-round. Our study complements these studies by providing the first detailed assessment of the temporal variability of surface bacterioplankton composition and abundance at the same site. By combining bacterioplankton weekly data obtained by flow cytometry and 16S rRNA amplicon sequencing every month and examining their relationships with other biotic and abiotic variables, we aimed to address the following questions: (i) what are the weekly to monthly dynamics of bacterioplankton in the shallow coastal waters of the central Red Sea? and (ii) what environmental factors drive the abundance and diversity patterns of major phylogenetic groups?

## Materials and Methods

### Sample Collection and Site Description

Seawater samples were collected from the coastal waters of the Red Sea. The sampling station is located in the harbor area of King Abdullah University of Science and Technology (KAUST) in Thuwal, Saudi Arabia (22°18′23.20″ N, 39°6′10.71″ E). Measurements of physicochemical variables and sample collection were performed from January 2016 to December 2016 every week. Temperature, salinity, and dissolved oxygen (DO) concentration were measured at the surface water using a multiprobe system (556 MPS YSI probe). Nine liters of water were collected every week in acid-cleaned polycarbonate bottles. For molecular bacterial community analysis, an extra 5 l of water was collected in the same way every month.

Oxygen saturation, which represents the oxygen concentration at equilibrium with the atmosphere at the temperature and salinity observed, was computed using the equation of Benson and Krause (1984), and apparent oxygen utilization (AOU) was calculated as the difference between the oxygen saturation and the measured dissolved oxygen.

### Chlorophyll Size Fractionation

Concentrations of size-fractionated chlorophyll *a* (Chl *a*) were measured after sequential filtration of 200 mL water samples through polycarbonate filters (IsoporeTM membrane filters) with pore sizes of 20, 2 and 0.2 μm. The filters were kept at −80°C until analysis. Chl *a* was extracted in 90% acetone for 24 h in the dark at 4°C. A Turner Trilogy fluorometer calibrated with a chlorophyll a standard (from *Anacystis nidulans*, Sigma 167 Aldrich) was used to measure Chl *a* using the acidification method. Total Chl *a* was calculated by summing the three fractions.

### Inorganic Nutrients

Samples for dissolved inorganic nutrient analyses were filtered through pre-burned GFF Whatman filters, and filtrates were stored frozen at −20°C in sterile falcon tubes until analysis. Nitrate (NO_3_^−^), nitrite (NO_2_^−^), phosphate (PO_4_^3−^), and silicate (SiO_2_) were analyzed on a Bruan and Luebbe® Autoanalyzer following Grasshoff et al. (1999) for the automated analysis in the segmented flow (Grasshoff et al., 1999). Limits of quantification were 0.2, 0.05, 0.01, and 0.02 μmol L^−1^ for NO_3_^−^ and NO_2_^−^, PO_4_^3−^ and SiO_2_, respectively. All standards were prepared with a nutrient-free artificial seawater matrix in acid-washed material. Nutrient data for January are not available as the samples were taken from February on.

### DOC and DON Concentration

Samples for dissolved organic carbon (DOC) and total dissolved nitrogen (TDN) were filtered through pre-burned GFF Whatman filters, acidified with H_3_PO_4_ to pH 1–2, and kept in the dark at 4°C until analysis by high-temperature catalytic oxidation (HTCO) in the laboratory on a Shimadzu TOC-L. All glass material was acid-cleaned and burned (450°C, 4.5 h). Consensus reference material of deep-sea carbon (42–45 μmol C L^−1^ and 31–33 μmol N L^−1^) and low carbon water (1–2 μmol C L^−1^), kindly provided by D. A. Hansell and Wenhao Chen (University of Miami), was for DOC and TDN concentration measurements to monitor the ultimate accuracy. Dissolved organic nitrogen (DON) concentrations were calculated by subtracting the dissolved inorganic nitrogen (DIN) from the TDN concentrations (DON = TDN-DIN), where DIN (μmol C L^−1^) = [NO_3_^−^] + [NO_2_^−^]. C:N molar ratios of the dissolved organic matter were calculated as the quotient DOC/DON in μmol L^−1^.

### Flow Cytometry: Bacterial Abundance, Cell Size, and Biomass

Bacterioplankton groups were analyzed on a FACSCanto flow cytometer with a blue 488 nm laser. For the determination of the Live cells, the fluorescent probes, SYBR Green I (Molecular Probes, Eugene, Oreg.) and propidium iodide (PI; Sigma Chemical Co.), were used for the double staining of nucleic acids. Both stains were added simultaneously to the non-fixed sample and left 10 to 15 min for staining in the dark. Cells with damaged membranes will be penetrated by the live stain (SYBR green) and the dead stain (PI), though the dead stain supposedly binds more strongly to DNA. The actively respiring cells were distinguished by adding 5-cyano-2,3-di-(p-tolyl) tetrazolium chloride (CTC) stain to a non-fixed sample. The redox dye 5-cyano-2,3-ditolyl tetrazolium chloride (CTC) is reduced intracellular in respiring cells to an insoluble, fluorescent precipitate. This product is detectable and quantifiable by flow cytometry in individual cells.

Two other groups of heterotrophic bacteria were analyzed from samples fixed with 1% paraformaldehyde plus 0.05% glutaraldehyde and distinguished based on their relative green fluorescence (FL1, 530 nm) as a proxy for nucleic acid content, referred to as high nucleic acid (HNA) and low nucleic acid (LNA) bacteria. The fixed samples were stained with SYBR-Green I (Molecular Probes) and incubated for 10 min in the dark. Samples were run for 10,000 events at low speed (15–20 mL min^−1^). The side scatter (SSC) data were used to estimate the individual cell size. One μm fluorescent beads (Molecular Probes) were used as a standard to obtain relative units (ru) of the intensity of the green fluorescence and side scatter signals. Volume was calculated from the side scatter measurements by assuming a spherical shape for all the cells, first converted to diameter using an empirical calibration (Calvo-Díaz and Morán, 2006; Huete-Stauffer et al., 2016). The cell size obtained was converted into biomass using Gundersen et al. (2002) conversion: fg C cell^−1^ = 108.8 × cell size^0.898^ and converted into bacterial biomass (BB, μmol C L^−1^).

### DNA Extraction and Amplicon-Based Next-Generation Sequencing

For total DNA extraction, 5 liters of water was filtered using Sterivex 0.22 μm filters, and the filters were stored at −80°C until DNA extraction. Power® Soil DNA Isolation Kit (MoBio Laboratories, Carlsbad, CA) was used for genomic DNA extraction from the stored filter with slight modifications to the protocol by adding lysozyme and achromopeptidase to the lysis buffer (Ansari et al., 2015). DNA quality was assessed using 260/280 nm and 260/230 nm ratios with a NanoDrop ND-1000 Spectrophotometer (NanoDrop Technologies, US). To perform amplicon next-generation sequencing, the total DNA was amplified for the V4–V5 region of 16S rRNA genes with the primer sequences for 515F and 907R (Caporaso et al., 2011; Ansari et al., 2015; Parada et al., 2016) modified with the Illumina adaptors: forward primer Illum_515F (5′-TCGTCGGCAGCGTCAGATGTGTATAAGAGACAGGTGYCAGCMGCCGCGGTAA-3′) and reverse primer Illum_907R (5′-GTCTCGTGGGCTCGGAGATGTGTATAAGAGACAGCCCCGYCAATTCMTTTRAGT-3′). The underlined regions of the primer sequences target the 16S rRNA gene.

### 16S rRNA Gene Amplification and Illumina MiSeq Sequencing

Illumina MiSeq was used for next-generation sequencing of 16S rRNA gene amplicons. PCR amplification was performed in a Gene Amp PCR-System 9,700 (Applied Biosystems, USA) in a total volume of 25 μl containing 2.5 μl 10X PremixF and 0.5 units of AccuPrime™ Taq DNA Polymerase High Fidelity (Life Technologies, USA), 0.4 μM of each primer, and 10 ng template DNA. Thermal cycling conditions were as follows: initial denaturation at 95°C for 3 min, and 25 cycles at 95°C for 30 s, 55°C for 30 s, and 72°C for 30 s, with a final extension at 72°C for 5 min. Following amplification, 2 μl of PCR product was used to confirm successful amplification using agarose gel (1%) electrophoresis. The PCR cleanup of the product was performed using AMPure XP beads. As described in the MiSeq Reagent Kit Preparation Guide (Illumina, USA), the purified mixture was used in the index PCR to attach dual indices and Illumina sequencing adapters using the Nextera XT index kit. Later, the PCR products were purified and quantified, and different samples were pooled and submitted to the KAUST core lab for sequencing.

### Sequence Analysis and Data Processing

Raw read sequences were quality filtered and trimmed using Trimmomatic v0.32 (Bolger et al., 2014) to remove adapter sequences and leading and trailing bases with a quality score below 20, less than 50 base-pairs (bp) long as well as reads with an average per-base quality of 20 over a 4-bp window. This preprocessing step also included a mapping-based step to remove reads of the internal standard PhiX using BBmap v37.44.^1^ At each stage, the quality of read sequences was assessed using FASTQC.^2^

The resulting high-quality paired-end reads for each dataset were then independently error-corrected with Bayeshammer algorithm implemented in SPAdes v3.9.0 (Nurk et al., 2017). The error-corrected reads were then analyzed with MOTHUR v1.3.95 (Schloss et al., 2009) using the standard protocols. A total of 847,501 unique reads from a total of 2,223,751 read sequences (200–301 bp in length and no ambiguities) were in the final dataset of the 12 samples used for the downstream analyses (alignment against Greengene-based “core_set_aligned.imputed.fasta” reference, chimera detection using UCHIME, removal of chloroplast sequences, and preclustering). The final dataset was taxonomically assigned against the SILVA database version 119 and accompanying taxonomy as implemented in MOTHUR. Finally, sequences were clustered at 97% sequence identity cut off to estimate species richness in the samples (rarefaction curves) and derive diversity indices for the full dataset and subsampling of each dataset down to the number of sequences present in the smallest sample (in January with 107,833 sequences). Correlation analyses were done in R using the R package “corrplot” based on the square-root transformed OTU abundances, and environmental parameters averaged for each month. Bray–Curtis similarities were calculated from square-root transformed relative abundance data. Multidimensional scaling (MDS) plots were generated from bootstrapped Bray–Curtis similarities using Primer-E software, version 7.

## Results

### Environmental Variables

**Figure 1:**
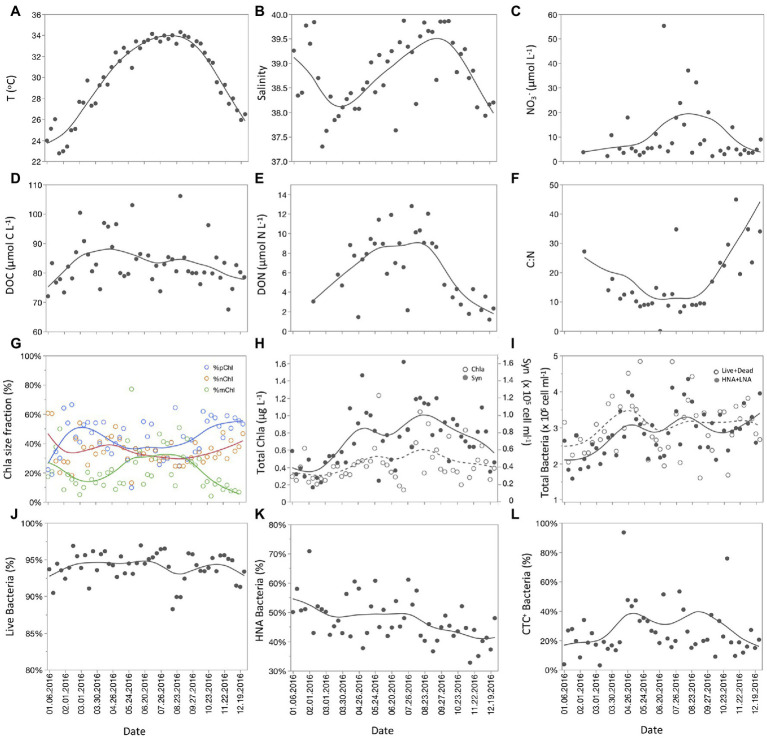
Temporal distribution of different environmental variables and bacterioplankton groups at the Red Sea coastal site: **(A)** temperature (°C); **(B)** salinity; **(C)** nitrate concentration (μmol L^−1^); **(D)** DOC concentration (μmol L^−1^); **(E)** DON concentration (μmol L^−1^); **(F)** DOM C:N ratios; **(G)** the percentage of the different size fractions pico-Chl *a* (blue), nano-Chl *a* (orange), and micro-Chl *a* (green); **(H)** total Chl *a* concentration (μg L^−1^; empty circles, dashed line) and *Synechococcus* abundance (cells mL^−1^; full circles, solid line); **(I)** total heterotrophic bacterial abundance (cells mL^−1^) considering the sum of live and dead bacteria (empty circles, dashed line) and the sum of HNA and LNA bacteria (full circles, solid line); **(J)** percentage of HNA bacteria (%); **(K)** percentage of Live bacteria (%); **(L)** percentage of actively respiring bacteria or CTC^+^ (%). The smooth curves fitted in each plot are weighted functions using the cubic spline with a lambda of 0.05 and standardized X values, generated on JMP Pro 16 software.

The coastal area at KAUST harbor displayed high temporal variability for most of the measured environmental variables. Temperature averaged 30.0 ± 3.5°C with a minimum of 22.8°C in January and a maximum of 34.3°C in August. Temperature remained higher than 34°C between mid-July and mid-September, when it began to decrease again (Figure 1A). Salinity changed only moderately, averaging 38.8 ± 0.7, ranging from 37.3 in late February to 39.9 in July. The seasonal pattern showed consistently low values (<38) from late February to late April, with higher values (>39.5) between mid-July and mid-October (Figure 1B). Dissolved oxygen concentrations (Supplementary Figure S1) averaged 5.64 ± 0.79 mg L^−1^ and ranged from 4.56 mg L^−1^ (July) to 7.17 mg L^−1^ (January), decreasing by 0.17 mg L^−1^ for each 1°C increase in temperature (*r* = 0.61, *p* < 0.0001, *n* = 50). AOU averaged 61.6 μmol Kg^−1^ and showed clear seasonality, with values ranging from −110.4 μmol Kg^−1^ (in January) to −23.1 μmol Kg^−1^ (in July).

Monthly mean nitrate values ranged from 3.7 μmol L^−1^ (February) to 22.3 μmol L^−1^ (August); highest values were recorded in late June (55.3 μmol L^−1^), with a few other values higher than 10 μmol L^−1^ scattered throughout the year (Figure 1C). Nitrite concentration averaged 0.15 ± 0.06 μmol L^−1^ and showed less variability than nitrate, yet a smooth seasonality with a minimum value in March (0.05 μmol L^−1^) that increased toward summer (up to 0.25 μmol L^−1^) was found. Phosphate concentrations averaged 0.12 ± 0.06 μmol L^−1^ (Supplementary Figure S1) and showed minimum values in March (0.03 μmol L^−1^) and increased toward summer. The highest values were measured in December for phosphate (0.25 μmol L^−1^). Silicate ranged from 2.9 (February) to 19.4 μmol L^−1^ (August) and showed a similar seasonality pattern as nitrate (Figure 1C; Supplementary Figure S1). Indeed, both nutrients were strongly and positively correlated (*r* = 0.97, *p* < 0.0001, *n* = 40).

### DOC, DON, and C:N Ratios

DOC concentrations averaged 83.3 ± 8.0 μmol C L^−1^ and ranged from 67.5 (November) to 106.1 (August) μmol C L^−1^ (Figure 1D). DOC seasonal patterns showed a maximum between March and April that coincided with a minimum in salinity. DON averaged 6.6 ± 3.4 μmol N L^−1^ and ranged from 1.16 (December) to 12.8 (August). A clear maximum with values above 10 μmol N L^−1^ was observed between June and August, when the highest temperatures were measured (Figure 1E). DON increased significantly with temperature (*r* = 0.63, *p* < 0.0001, *n* = 39). The C:N ratio of dissolved organic matter (DOM) averaged 19.3 ± 15.5, with values below 10 between May and August (Figure 1F), suggesting its bioavailability increased during the warmest months.

### Total and Size-Fractionated Chlorophyll a

Total chlorophyll *a* averaged 0.45 ± 0.23 μg L^−1^ and ranged from 0.14 (July) to 1.23 (May) μg L^−1^ (Figure 1H). Total Chl *a* variability was highest in summer, but seasonality was not evident. The small (pico- and nano-) size-classes contribution to total values averaged 44 ± 13% and 35 ± 10%, respectively, and showed no clear seasonality. The proportion of picophytoplankton was dominant throughout the year, ranging from 27 to 56%, but decreased in summer when the proportion of microphytoplankton increased to more than 40% (Figure 1G). Total Chl *a* was correlated positively with temperature (*r* = 0.33, *p* = 0.02, *n* = 50), silicate (*r* = 0.37, *p* = 0.02, *n* = 40) nitrate (*r* = 0.36, *p* = 0.03, *n* = 40), and DON (*r* = 0.42, *p* = 0.01, *n* = 39).

### Bacterioplankton Abundance and Single-Cell Physiological Structure

The dynamics of the different groups of bacteria identified are presented in Figures 1I–L. Total heterotrophic bacteria abundance can be calculated as the sum of LNA and HNA cells or as the sum of live and dead cells, with both represented in Figure 1I. Two peaks were detected, in April–May and August (3.99 and 4.35 × 10^5^ cells mL^−1^, respectively, using LNA + HNA and live+dead values). The percentage of live bacteria was above 90% for most of the year and showed slight fluctuations (Figure 1J). The percentage of HNA cells varied from around 35% (November to December) to over 60% (July; Figure 1K). CTC+ cells were consistently less abundant, but varied widely from 4% (March) to around 50% (May; Figure 1L). Cyanobacteria were almost exclusively represented by *Synechococcus*, as *Prochlorococcus* was found in very low numbers and only detected in four samples, two in January and one each in February and March (1.1 × 10^4^, 5.79 × 10^3^, 1.3 × 10^4^, and 4.7 × 10^3^ cells mL^−1^, respectively). *Synechococcus* abundance showed a similar pattern to that of total heterotrophic bacteria (Figures 1H,I), with two maxima in April–May and August (1.20 and 1.46 × 10^5^ cells mL^−1^, respectively). Both *Synechococcus* and heterotrophic bacteria abundances decreased in June and July. Abundance of HNA, *Synechococcus*, and CTC+ cells was positively correlated with temperature and total Chl *a* (Table 1). Actively respiring bacteria (CTC+) was the group most affected by environmental variables and showed significant positive correlations with temperature, DOC, total Chl *a* (Table 1) but a negative correlation with phosphate concentration (rho = −0.33, *p* = 0.04, *n* = 40).

**Table 1:** Spearman correlation coefficients (rho) between bacterial population abundances (cells mL^−1^) and selected environmental and nutritional factors: Temp (temperature, °C), salinity, DO (dissolved oxygen, mg L^−1^), DIN (dissolved inorganic nitrogen, μmol L^−1^), phosphate (μmol L^−1^) DOC (dissolved organic carbon, μmol L^−1^), DON (dissolved organic nitrogen, μmol L^−1^), and Chl *a* (μg L^−1^) at the Red Sea coastal site.

Spearman’s rho correlations								
Bacterial groups	Temp	Salinity	DO	DIN	Phosphate	DOC	DON	Chl *a*
*Synechococcus*	0.63***	0.22	−0.48***	−0.04	−0.04	0.10	0.10	0.51***
Live	0.13	−0.06	−0.07	−0.16	−0.02	−0.05	−0.10	0.41***
LNA	0.27	0.06	−0.28*	−0.20	0.20	0.10	0.30	0.36**
HNA	0.31*	0.06	−0.21	0.04	0.02	0.01	−0.01	0.42***
CTC+	0.46***	0.01	−0.25	−0.13	−0.33*	0.37**	0.02	0.34*
N	50	50	50	40	40	49	39	50

* *p* *≤ 0.5;*

** *p* *≤ 0.01;*

*** *p* *≤ 0.001*.

Autotrophic and heterotrophic bacteria showed seasonal variations in their respective cell sizes (Supplementary Figure S2). The cell size of *Synechococcus* varied from 0.054 to 0.161 μm^3^, while the cell size of the average heterotrophic bacterium ranged from 0.040 to 0.054 μm^3^, which was due to the different temporal variability of LNA and HNA cells. HNA cells were consistently larger (0.051 to 0.077 μm^3^) than their LNA counterparts (0.028 to 0.040 μm^3^) year-round The cell size of LNA, HNA, and *Synechococcus* increased with increasing temperature from January to June and then remained constant until it decreased again from October to December as temperature decreased. HNA cell size showed a positive relationship with temperature (*r* = 0.55, *p* = 0.0001, *n* = 50) and DON concentration (*r* = 0.36, *p* = 0.026, *n* = 39), while LNA cell size was positively correlated only to temperature (*r* = 0.62, *p* = 0.0001, *n* = 50). *Synechococcus* cell size showed a positive correlation with DIN (rho = 0.38, *p* = 0.016, *n* = 39) and temperature (rho = 0.59, *p* = 0.0001, *n* = 47).

The total biomass of heterotrophic bacteria (LNA + HNA) ranged from 2.06 to 6.42 μg C L^−1^, which was always higher than that of *Synechococcus* (0.38–4.00 μg C L^−1^). Consequently, the ratio of autotrophic to heterotrophic bacterial biomass ranged from 0.1 to 0.8, with higher values in July and lower in February. However, as expected from the changes in abundance, both heterotrophic bacteria biomass (*r* = 0.51 *p* < 0.0001, *n* = 50) and *Synechococcus* biomass (*r* = 0.61, *p* < 0.0001, *n* = 50) were significantly correlated with temperature.

### Bacterial Community Structure

**Figure 2:**
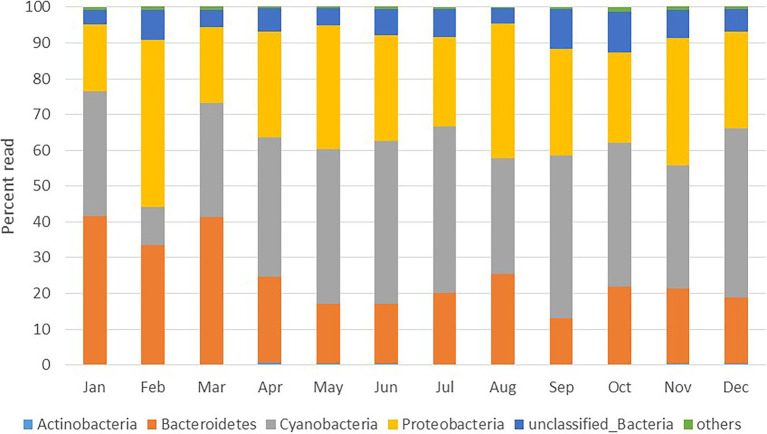
Monthly changes in bacterial community composition at the phylum level expressed as percent of total reads.

**Figure 3:**
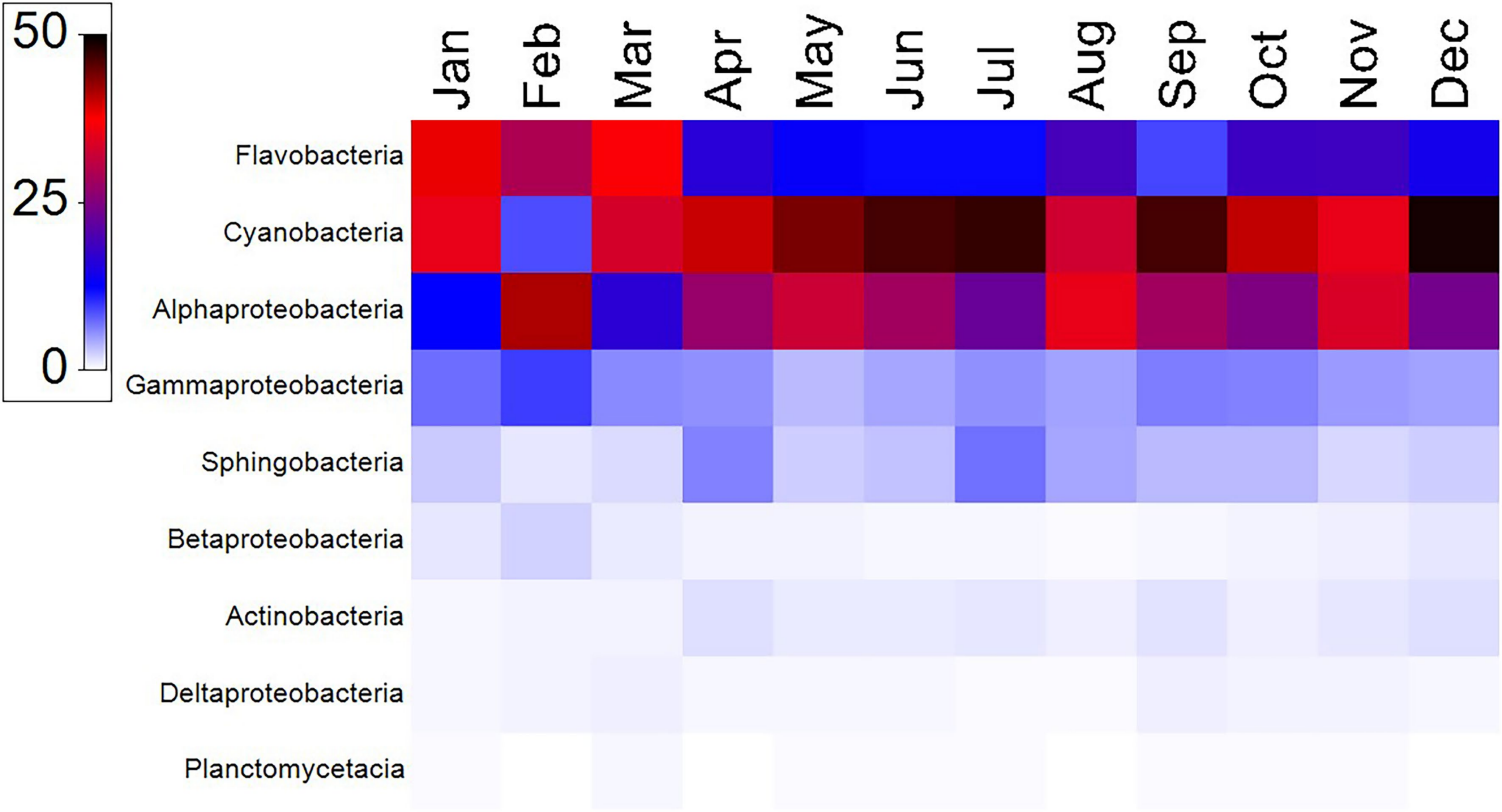
Heat map displaying the relative abundances (color code represents the percent relative abundances) and distribution of highly represented bacterial taxa at the class level at the coastal site of the Red Sea.

A total of 1,888,244 16S rRNA gene sequences were obtained after quality control for all 12 samples collected in the sampled year (Supplementary Table S1). The alpha diversity measures show that the maximum diversity was found from September to November (Supplementary Table S1). The majority of the sequences obtained belonged to the phyla Cyanobacteria (8–49%, mean = 38%), Proteobacteria (21–40%, mean = 34%) and Bacteriodetes (13–42%, mean = 24%; Figure 2). Among Cyanobacteria, subsection I (8–48.75%) was the dominant clade, mainly *Synechococcus* (8–48.75%). Within the phylum Proteobacteria, Alphaproteobacteria (12–35%) were the most abundant class, followed by Gammaproteobacteria (3–7%) and Betaproteobacteria (0.2–2%). Within Bacteriodetes, the most abundant classes were Flavobacteria (8.7–38.7%), Sphingobacteria (0.6–5.1%), Cytophagia (0.02–3.25%), and Bacteroidia (0.01–0.04%). Figure 3 shows the heat map of the nine dominant classes. The proportion of Alphaproteobacteria was low in January and March when Flavobacteria predominated in abundance.

**Figure 4:**
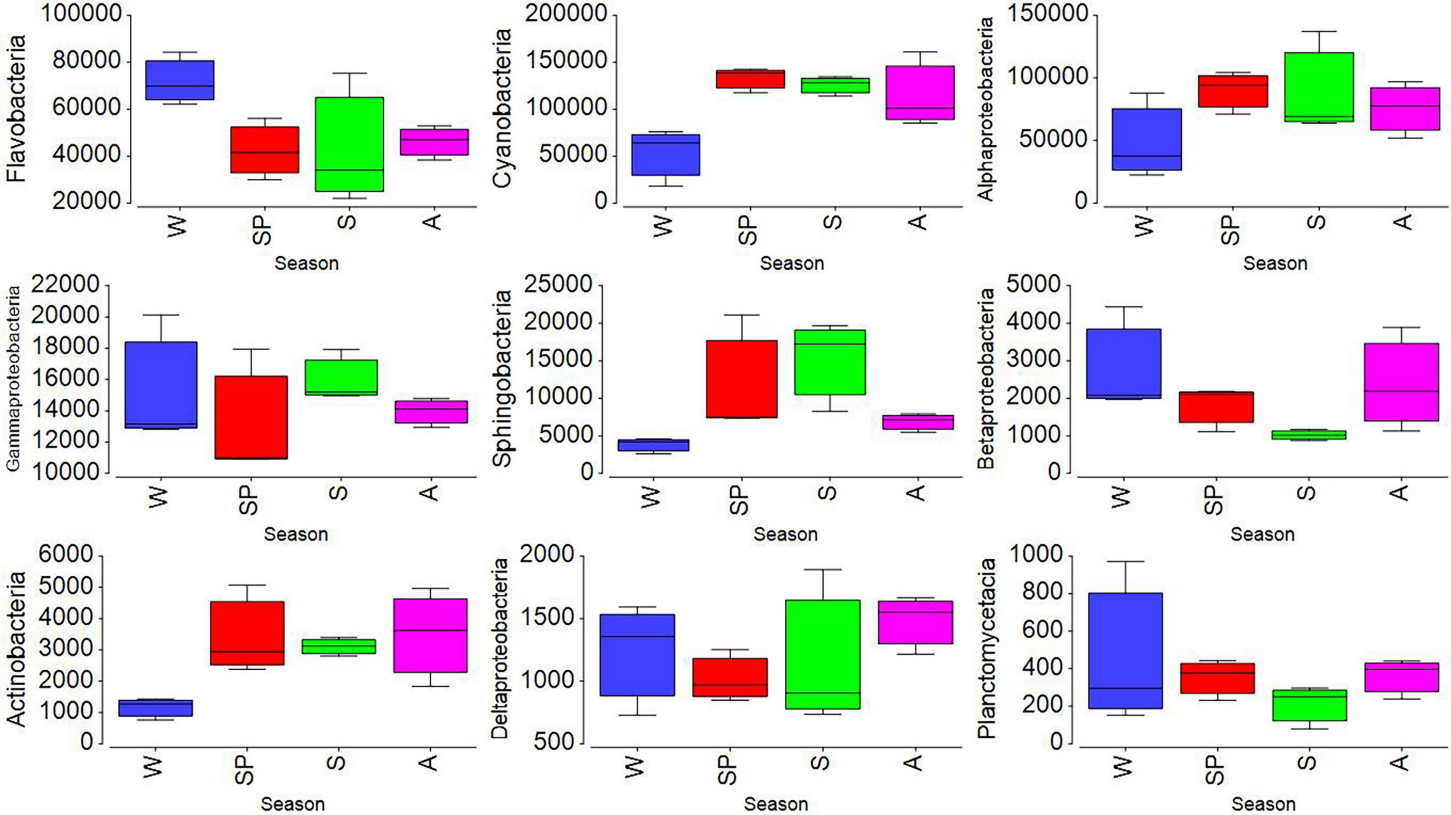
Seasonal variations of selected dominant taxonomic class (cells mL^−1^) obtained by multiplying their relative abundances by total bacteria abundance obtained from flow cytometry analysis. Here, symbols represent W (winter), SP (spring), S (summer), and A (autumn).

**Figure 5:**
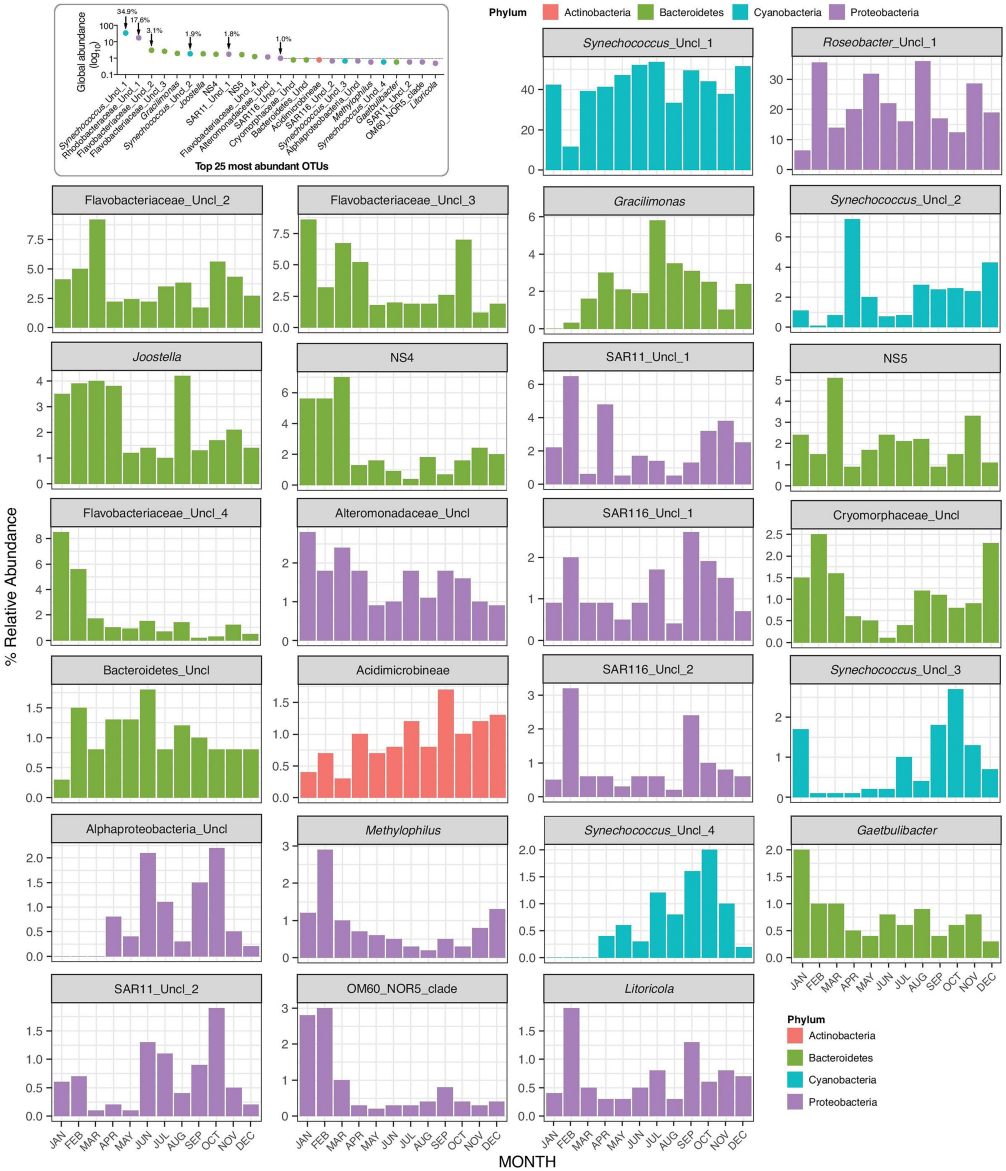
Monthly changes in the relative abundances of the top 25 OTUs present at the Red Sea coastal site.

To further analyze the seasonality of the different classes, their relative percentages were multiplied by the total heterotrophic bacterial abundances obtained from flow cytometry. The mean seasonal abundances of the nine major classes are shown in Figure 4. The values obtained were found to be significant by using two-way ANOVA (*p* < 0.001). Alphaproteobacteria, Gammaproteobacteria, and Sphingobacteria increased in summer, while Flavobacteria and Betaproteobacteria decreased (Figure 4). Cyanobacteria showed the lowest value in winter (February), consistent with the low abundances of *Synechococcus* detected by flow cytometry in that month (cf. Figures 2, 5). At the family level, Synechococcaceae (8–47%), Flavobacteriaceae (5–18%), Rhodobacteraceae (6–26%), and Pelagibacteraceae (1–5%) dominated. The highest abundance of Flavobacteriaceae (18%) was found in January, coinciding with the lowest abundance of Rhodobacteraceae (6%). The highest relative abundance of Rhodobactereceae was found in August (31%), while the abundance of Flavobacteriaceae was 11%. A negative relationship was found between the percentage of Flavobacteriaceae and temperature (*r* = −0.58, *p* = 0.04, *n* = 12).

OTU based analysis of the sequences revealed that *Synechococcus* varied from 33 to 49% of the total OTUs, except in February when they accounted for only 11% of total reads (Figure 5). The percentage of *Synechococcus* was multiplied by the abundance of bacteria (including *Synechococcus*) from flow cytometry to obtain the abundance of *Synechococcus* from sequence analysis (Supplementary Figure S3). Although the abundance of *Synechococcus* cells was estimated to be twice as high by sequencing compared to flow cytometry counts (2.82 × 10^4^–2.22 × 10^5^), the seasonal trend was the same for both methods (*r* = 0.82, *p* = 0.001, *n* = 12).

**Figure 6:**
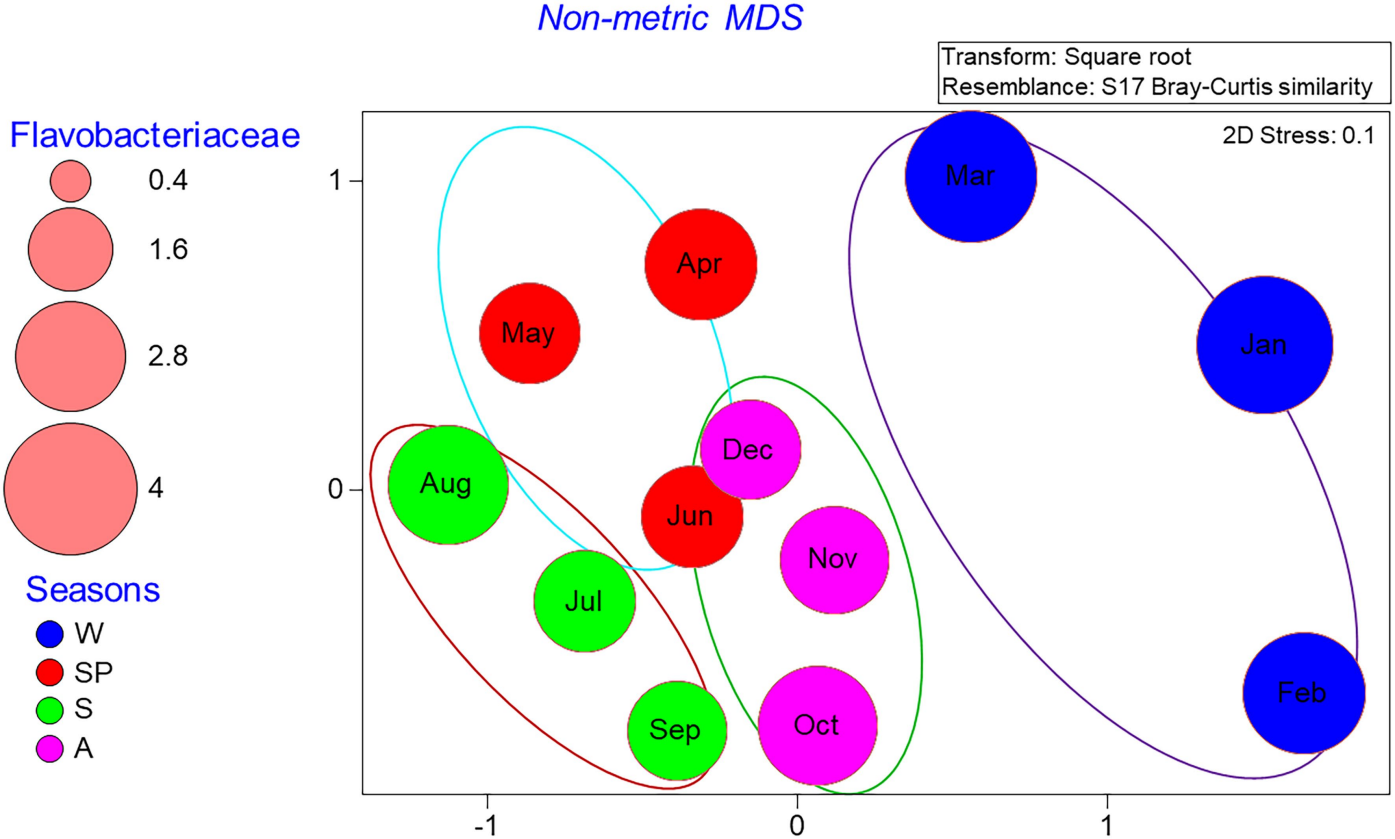
Distribution of monthly bacterial communities in a nMDS plot in the Red Sea coastal site. The Venn diagram shows cluster overlaps within different months according to seasons. The size of the bubbles indicates the percentage of Flavobacteriaceae cells in each month of 2016, while seasons are represented by W (winter), SP (spring), S (summer), and A (autumn).

**Figure 7:**
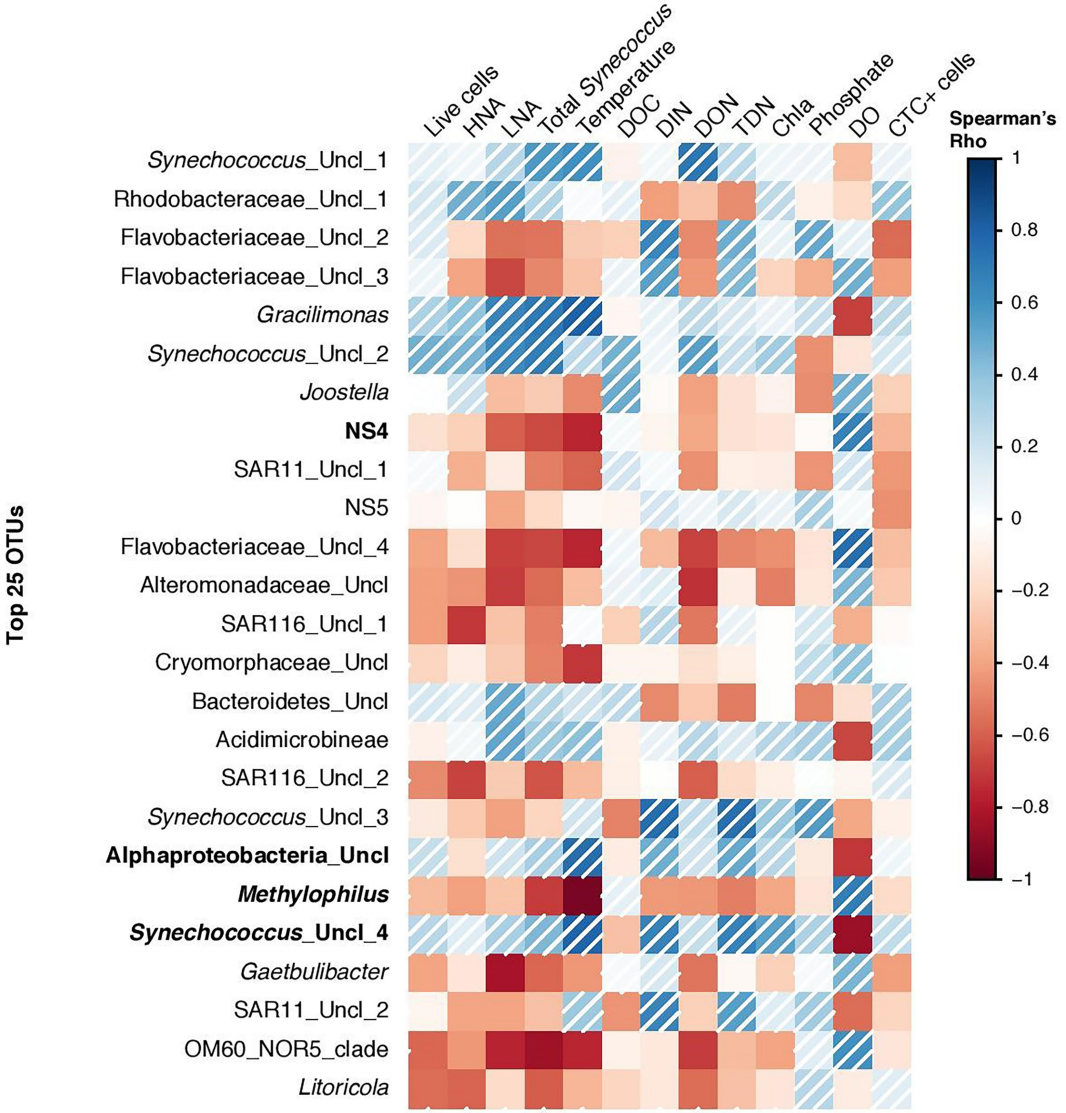
Spearman correlation coefficients between the top most abundant 25 OTUs and bacterial and environmental factors at the coastal site (in the Red Sea). Bold OTUs represents new genera or group of bacteria showing highest Spearman correlations with temperature or oxygen.

Non-metric multidimensional scaling (NMDS) analysis of the relative abundance of bacterial groups at the genus level showed clear community differentiation by seasonality. Winter and summer samples were clustered separately from spring and fall samples (Figure 6). The autumn and spring samples were also separated, except for the June and December samples, which were close to each other. PERMANOVA analysis also confirmed the significant differences between OTU composition in different seasons (*p* = 0.03). Spearman correlation analysis showed that bacterial diversity was mostly correlated with temperature, dissolved oxygen, DIN, and DON (Figure 7). Alphaproteobacteria, *Synechococcus*, and *Gracilimonas* (Balneolaceae) were positively correlated with temperature and negatively correlated with DO, while NS4 (Flavobacteria) and *Cryomorphaceae* were negatively correlated with temperature and positively correlated with DO (Figure 7).

## Discussion

The study site on the coast of the central Red Sea depicted clear seasonal patterns for many of the environmental variables. In addition to a 11.5°C change in temperature and a 2.6 increase in salinity, concentration of nitrate was particularly high during summer months. Other variables affected by seasonality in the coastal waters of the Red Sea were silicate, DOM and DO concentrations. Since there is strong stratification year-round in the deeper waters nearby, the above-mentioned inorganic nutrients could originate from Aeolian dust deposition (mostly occurring due to wind-blown dust from the surrounding desert; Singh et al., 2008). Recent studies have reported a clear seasonal pattern of dust emission and deposition over the Arabian Red Sea coast, with the highest inputs occurring between June and August (Anisimov et al., 2017). These authors also assessed the mineralogical composition of dust and concluded that quartz (a source of dissolved silicate) was the most abundant mineral. The fact that nitrate and silicate were strongly correlated and followed the same seasonal pattern with maxima between June and August (Figure 1C; Supplementary Figure S1) suggests that their seasonal changes could be explained by dust deposition (Raitsos et al., 2015).

Phosphorus was limiting year-round, and the competition between heterotrophic bacteria and phytoplankton for phosphate might have played a role in the generally low phytoplankton biomass despite high nitrogen loading. Silva et al. (2019) have shown that heterotrophic bacteria took up phosphate in ca. 80% of the incubations without protists and phytoplankton carried out over slightly more than 1 year. Notwithstanding Chl *a* values seldom exceeding 1 μg L^−1^, high silicate concentrations in summer were correlated with a higher proportion of microphytoplankton (probably due to the presence of silicate-requiring diatoms), which is inconsistent with the frequent association of warmer temperatures with more oligotrophic conditions in tropical and temperate waters and the subsequent increase in the contribution of smaller-sized phytoplankton (Rasconi et al., 2017).

Although higher primary productivity and oxygen production would be expected with higher Chl *a*, increases in phytoplankton standing stocks were typically small and short-lived (Figure 1H) hence unable to obscure the drop in DO caused by summer warming (Supplementary Figure S1). However, changes in AOU indicate that the utilization of oxygen (i.e., respiration) also peaked between July and September. Even though AOU values were always negative (indicating that phytoplankton oxygen production exceeded oxygen consumption), there was a clear increase in respiration by heterotrophic bacteria during the warmest months. Accordingly, AOU significantly correlated with increasing temperature (*r* = 0.65, *p* < 0.0001, *N* = 49) and bacterial cell numbers (HNA + LNA *r* = 0.31, *p* = 0.03, *N* = 49).

The weekly variability in the abundance and cell properties of bacterioplankton of this study complements the same high-frequency observations obtained 1 year later, in 2017 (Sabbagh et al., 2020). The changes in abundance of total heterotrophic bacteria, with maxima in spring and late summer and minima in winter, are consistent with results elsewhere (Šantić et al., 2012; Cram et al., 2015; He et al., 2017), including the tropical region (Zhao et al., 2016; Sabbagh et al., 2020). HNA bacteria predominate in meso- to eutrophic environments, while LNA bacteria are generally dominant in oligotrophic conditions (Zubkov et al., 2004; Morán et al., 2007). Although LNA and HNA cells did not show a clear seasonal pattern, both groups tended to be evenly distributed in the first half of the year (ca. 50% each), while LNA slightly predominated between September and the end of the year (Figure 1K). This coincided with a decline in microphytoplankton as well as Chl *a* (Figure 1H) and nitrate (Figure 1C), after these variables peaked in August.

The highest peak in DOC concentration found between March and April coincided with the highest proportion of picophytoplankton relative to total phytoplankton biomass measured as Chl *a*, and a clear increase in *Synechococcus* cell numbers (Figures 1D,G,H). This puts forward for consideration that a portion of the available DOC during this period could be the result of primary production of small autotrophic cells. Moreover, we found a positive and significant correlation between concentrations of DOC and abundance of *Synechococcus* when considering samples taken during March and April (*p* = 0.03, *r* = 0.71, *N* = 9), when *Synechococcus* first peaked. In contrast, the availability of DOC during the summer months could be related to the primary production mediated by a mix of both large autotrophic cells (i.e., diatoms), which were more abundant in July and August (up to 50.8% of the total; Figure 1G), and *Synechococcus*, which were peaking for a second time (Figure 1H). Although there is no significant statistical support for the months of July and August (most probably due to the concomitant autotrophic DOC production and heterotrophic DOC consumption in natural waters), the observed patterns seem to indicate that DOC, likely produced by small autotrophic cells, may have triggered the increase in absolute heterotrophic bacterial abundance (Figure 1I) and the contribution of actively respiring bacteria (CTC+, Figure 1L). We found a significant and positive correlation between DOC concentration and total heterotrophic bacterial abundance (Live + Dead) when considering a gap time of 2 weeks (*p* = 0.05, *r* = 0.28, *N* = 49), that supports this hypothesis. Total heterotrophic bacterial biomass was 14% higher during the summer peak than in March–April. However, other microbial processes in the food web [e.g., protistan grazing and viral infections, (Sabbagh et al., 2020)] and physicochemical transformations (e.g., photobleaching) may also influence the concentration and lability of available DOC. These results are supported by a previous study that showed high DOC uptake rates from February to May in monthly incubations of heterotrophic bacterioplankton, both in the absence and in the presence of grazers at the same study site (Silva et al., 2019).

Our results showed that HNA cells were consistently larger than LNA cells year-round, as is usually the case elsewhere (Huete-Stauffer et al., 2016; Liu et al., 2016), but in contrast to other reports in which LNA cells can occasionally reach or even exceed the size of HNA cells (Bouvier et al., 2007; Gomes et al., 2015; Morán et al., 2015). Cell size increased with increasing temperature up to 32°C in both groups, but with a lag of about 3 months (Supplementary Figure S2). LNA cell size increased when the temperature first rose in June/July, whereas HNA cell size increased after temperature remained high for 3 months (in September to October). These findings contradict recent results from temperate ecosystems (Fauteux et al., 2015; Morán et al., 2015; Huete-Stauffer et al., 2016) that report a general decrease in cell size with increasing temperature. Although the relationships between abundance, body size, and temperature are complex (Kingsolver and Huey, 2008), a universal ecological principle states that higher abundance of organisms should be associated with smaller individual size (White et al., 2007). Of note, previous studies showing a decrease in cell size with increasing temperature were performed in colder environments than the Red Sea. In contrast, the minimum temperature found in our study (23°C, Figure 1A) was even higher than the maximum temperature reached in this temperate site study (Morán et al., 2015).

*Synechococcus* cell size showed a similar seasonality, although relative maxima were also observed in March (Supplementary Figure S2). Therefore, the temperature-size rule (Forster et al., 2013) does not seem to explain our findings. However, our observations also suggest a higher nutritional status (DOM with relatively higher N content) of heterotrophs during the warmest season. DON values were generally elevated between June, July, and August (Figure 1E) when temperatures remained above 32°C (Figure 1A), resulting in lower C:N ratios and more labile DOM. Consistent with the argument that bacterial cell size also reflects activity (Gasol et al., 1995), a recent study at the same coastal site reported higher bacterial growth efficiency values in summer, which coincided with the lowest C:N ratios (Silva et al., 2019). The explanation for the reduction in cell size at higher temperatures could be due to metabolic advantages for organisms under warmer conditions (Daufresne et al., 2009). However, it remains debatable whether temperature can directly cause the temperature-size variability, as other factors like DOM lability and availability, oxygen diffusion, or size-selective predation could be more important players (Daufresne et al., 2009; Gardner et al., 2011; Huete-Stauffer et al., 2016). This appears to be the case in our study, where temperature and DON (indicative of more labile DOM compounds) were positively correlated with Chl *a*.

Moreover, the abundance of LNA, HNA and CTC+ cells were correlated significantly with total Chl *a*, further suggesting the role of organic substrates released by primary producers (Ni et al., 2015). The positive correlation between the abundances of CTC+ and HNA bacteria suggests that the most metabolically active cells belong mainly to the latter group throughout the year, as previously shown in Blanes Bay, in the Mediterranean Sea (Baltar et al., 2015) and Waquoit Bay in the Northwest Atlantic (Morán et al., 2011). The size increase of HNA cells with temperature (0.001 μm^3^ °C^−1^), which was more pronounced than that of LNA cells (0.0005 μm^3^ °C^−1^), also suggests that HNA bacteria were more active and responsive to changes in DOM quality, in agreement with differences in the respective specific growth rates (Silva et al., 2019).

Bacterial community composition was assessed monthly (rather than weekly), which still allowed us to assess key seasonal changes in diversity and compare them with other bacterioplankton properties. This study is one of the few to describe temporal changes in bacterial taxa throughout an annual cycle in the tropical coastal waters of the Red Sea, which were originally thought to be subject to less environmental variation compared to higher latitudes (Wang et al., 2015; Doherty et al., 2017). However, since there is already a temporal resolution discrepancy in the dataset (i.e., weekly variations in microbial abundance and ancillary environmental variables vs. monthly changes in 16S rRNA amplicons), we are afraid that we cannot explore different cause-effects time lags when it comes to changes in microbial community composition. Bacteroidetes, Cyanobacteria, and Proteobacteria were the predominant bacterial phyla, which showed variations in monthly abundances (Figure 2) similar to previous findings (Yin et al., 2013) in the surface waters of the oligotrophic South Pacific Subtropical Gyre (Ortmann and Ortell, 2014). Furthermore, our results revealed that Rhodobacteraceae, SAR11 and *Synechococcus* were the dominant groups throughout the year, except in February, thus showing that microbial community composition in tropical waters may occasionally fluctuate as much as in higher latitude regions. Similar to our results, Actinobacteria increased at other coastal sites in autumn, along with autumn-specific SAR11 members (Lindh et al., 2015; Bunse and Pinhassi, 2017). Overall, we observed a larger than expected microbial community succession throughout the year, with contrasting responses of different OTUs to biological and environmental parameters (Figure 7), especially temperature, DON, and DO.

From a seasonal perspective, winter microbial diversity differed from the remaining seasons (Figure 3) and was characterized by a relatively high abundance of Flavobacteria. The Shannon diversity index showed the highest value in February (2.95), with a large effective species number of 19,029, whereas the lowest index (2.01) was recorded in May. The combined use of flow cytometry and 16S rRNA sequencing can provide estimated absolute taxon abundances. In the case of *Synechococcus*, the higher abundance of cyanobacteria detected by amplicon sequencing, which virtually duplicated the flow cytometric counts could be due to the presence of multiple copies of the 16S rRNA gene (Moore et al., 2019). Certain groups can safely be assigned to either the LNA or the HNA flow cytometric groups, with little overlap between them. Particularly, the family Rhodobacterales and Flavobacteria would likely belong to the HNA group, whereas the SAR11 clade typically makes up a large fraction of LNA cells (Schattenhofer et al., 2011; Vila-Costa et al., 2012). Contrary to the expectation in warm and oligotrophic waters, SAR11 was not among the dominant members of the community, perhaps due to the near shore location of the study site and the unusual nutrient requirements (Carini et al., 2013).

In conclusion, this year-long study provides a comprehensive assessment of bacterioplankton abundance and diversity at, respectively, high and moderate temporal resolution and identifies the central players relevant in the ecosystem functioning and biogeochemistry of the coastal Red Sea. Remarkable changes in the abundance, physiological structure, and diversity patterns of planktonic bacteria in turn reflected changes in environmental variables, especially temperature, chlorophyll *a*, DOC concentration, and lability, which were not anticipated to fluctuate much in a tropical environment. Among the heterotrophs, Flavobacteriaceae, Rhodobacteraceae, and SAR11 were the groups most affected by these environmental factors, each with a distinct temporal signal in these shallow waters of the central Red Sea.

## Data Availability Statement

The raw 16S rRNA gene amplicon data can be found in the European Nucleotide Archive, project number PRJEB50961.

## Author Contributions

MIA, MLIC, and XAGM led the experiment design, set up the experiments, performed data analysis, and wrote the manuscript. MIA, MLIC, LS, MV, and TMH-S performed sampling and conducted most of the analyses. DKN performed the amplicon data analysis, interpreted the results, and wrote partly the section. XAGM conceived the research and contributed to interpretation. All authors contributed to the article and approved the submitted version.

## Funding

This research was supported by King Abdullah University for Science and Technology (KAUST) through the baseline funding provided to XAGM.

## Conflict of Interest

The authors declare that the research was conducted in the absence of any commercial or financial relationships that could be construed as a potential conflict of interest.

## Publisher’s Note

All claims expressed in this article are solely those of the authors and do not necessarily represent those of their affiliated organizations, or those of the publisher, the editors and the reviewers. Any product that may be evaluated in this article, or claim that may be made by its manufacturer, is not guaranteed or endorsed by the publisher.

## Footnotes

^1^ http://jgi.doe.gov/data-and-tools/bbtools/

^2^ http://www.bioinformatics.babraham.ac.uk/projects/fastqc/
