# Supplementary material for: High-Frequency Variability of Bacterioplankton in Response to Environmental Drivers in Red Sea Coastal Waters
Source: Front Microbiol. 2022 Mar 31;13:780530. doi: 10.3389/fmicb.2022.780530 (PMC9009512; doi:10.3389/fmicb.2022.780530)
Supplement: Supplementary file 1 [file Data_Sheet_1.DOCX]

**High-frequency variability of bacterioplankton in response to environmental drivers in Red Sea coastal waters**

Mohd Ikram Ansari^1, 2^*****, Maria Ll. Calleja^1, 3^, Luis Silva^1^, Miguel Viegas^1^, David K. Ngugi^1, 4^, Tamara M. Huete-Stauffer^1^, Xosé Anxelu G. Morán^1^

^1^ King Abdullah University of Science and Technology (KAUST), Division of Biological and Environmental Sciences and Engineering (BESE), Red Sea Research Center (RSRC), Thuwal 23955-6900, Saudi Arabia.

^2^ Department of Biosciences, Integral University, Kursi Road, Dasauli, Lucknow 226026 Uttar Pradesh, India

^3^ Department of Climate Geochemistry, Max Planck Institute for Chemistry (MPIC), Hahn-Meitner-Weg 1, 55128 Mainz, Germany

^4^ Leibniz Institute DSMZ - German Collection of Microorganisms and Cell Cultures, Braunschweig, Germany

*****Corresponding author

[mohd.ikram.ansari@gmail.com](mailto:mohd.ikram.ansari@gmail.com); xelu.moran@kaust.edu.sa

Figure S1: Temporal distribution of oxygen, phosphate and silicate at the Red Sea coastal site

Figure S2: Weekly variations of the cell size (µm^3^) of heterotrophic (HNA and LNA) and autotrophic bacteria (*Synechococcus*) at the coastal site of Red Sea in 2016.

Table S1. Sequencing details for the 16S amplicon data obtained after processing of the data in the original and the subsampled datasets with observed (sobs) and estimated (chao, ace) species diversity for the overall bacterial sequences present in different months of 2016.

Figure S3: Comparison of *Synechococcus* abundance from flow-cytometry (FCM, orange bars) and 16S sequencing (blue bars) obtained by multiplying the percent read from amplicon sequencing of *Synechococcus* (gray line) with total bacteria counts/abundance.
